# Diagnostic performance and generalizability of deep learning for multiple retinal diseases using bimodal imaging of fundus photography and optical coherence tomography

**DOI:** 10.3389/fcell.2025.1665173

**Published:** 2025-09-11

**Authors:** Xingwang Gu, Yang Zhou, Jianchun Zhao, Hongzhe Zhang, Xinlei Pan, Bing Li, Bilei Zhang, Yuelin Wang, Song Xia, Hailan Lin, Jie Wang, Dayong Ding, Xirong Li, Shan Wu, Jingyuan Yang, Youxin Chen

**Affiliations:** 1 Department of Ophthalmology, Peking Union Medical College Hospital, Chinese Academy of Medical Sciences, Beijing, China; 2 Key Laboratory of Ocular Fundus Diseases, Chinese Academy of Medical Sciences, Beijing, China; 3 Vistel AI Lab, Visionary Intelligence Ltd., Beijing, China; 4 Department of Nuclear Medicine, Peking Union Medical College Hospital, Chinese Academy of Medical Sciences, Beijing, China; 5 Department of Ophthalmology, The First Affiliated Hospital of Zhejiang University, Hangzhou, Zhejiang, China; 6 Department of Ophthalmology, Hunan Provincial People’s Hospital, Changsha, China; 7 Department of Ophthalmology, Peking University Third Hospital, Beijing, China; 8 Department of Ophthalmology, Guizhou Provincial People’s Hospital, Guiyang, China; 9 Key Lab of DEKE, Renmin University of China, Beijing, China; 10 Beijing Hospital, National Center of Gerontology, Institute of Geriatric Medicine, Chinese Academy of Medical Sciences, Beijing, China

**Keywords:** deep learning, diagnosis, fundus photography, optical coherence tomography, retinal disease

## Abstract

**Purpose:**

To develop and evaluate deep learning (DL) models for detecting multiple retinal diseases using bimodal imaging of color fundus photography (CFP) and optical coherence tomography (OCT), assessing diagnostic performance and generalizability.

**Methods:**

This cross-sectional study utilized 1445 CFP-OCT pairs from 1,029 patients across three hospitals. Five bimodal models developed, and the model with best performance (Fusion-MIL) was tested and compared with CFP-MIL and OCT-MIL. Models were trained on 710 pairs (Maestro device), validated on 241, and tested on 255 (dataset 1). Additional tests used different devices and scanning patterns: 88 pairs (dataset 2, DRI-OCT), 91 (dataset 3, DRI-OCT), 60 (dataset 4, Visucam/VG200 OCT). Seven retinal conditions, including normal, diabetic retinopathy, dry and wet age-related macular degeneration, pathologic myopia (PM), epiretinal membran, and macular edema, were assessed. PM ATN (atrophy, traction, neovascularization) classification was trained and tested on another 1,184 pairs. Area under receiver operating characteristic curve (AUC) was calculated to evaluated the performance.

**Results:**

Fusion-MIL achieved mean AUC 0.985 (95% CI 0.971–0.999) in dataset 2, outperforming CFP-MIL (0.876, *P* < 0.001) and OCT-MIL (0.982, *P* = 0.337), as well as in dataset 3 (0.978 vs. 0.913, *P* < 0.001 and 0.962, *P* = 0.025) and dataset 4 (0.962 vs. 0.962, *P* < 0.001 and 0.962, *P* = 0.079). Fusion-MIL also achieved superior accuracy. In ATN classification, AUC ranges 0.902–0.997 for atrophy, 0.869–0.982 for traction, and 0.742–0.976 for neovascularization.

**Conclusion:**

Bimodal Fusion-MIL improved diagnosis over single-modal models, showing strong generalizability across devices and detailed grading ability, valuable for various scenarios.

## 1 Introduction

Retinal diseases are one of the leading causes of irreversible blindness worldwide, including age-related macular degeneration (AMD) and diabetic retinopathy (DR) (Bourne et al., 2013). Color fundus photography (CFP) and optical coherence tomography (OCT) are widely used imaging modalities, with CFP providing en-face views and OCT offering high-resolution cross-sectional scans. Although deep learning (DL)-based screening or diagnosis of retinal diseases using CFPs has been extensively investigated, CFP alone may be insufficient for detecting certain retinal conditions due to limitations in information dimensions (Ting et al., 2019; Schmidt-Erfurth et al., 2018). For instance, the Atrophy-Traction-Neovascularization (ATN) classification system for myopic maculopathy emphasizes both en-face and cross-sectional changes of the retina, which requires the application of CFP and OCT technology for better grading (Ruiz-Medrano et al., 2019). Other retinal diseases can also rely on such enhanced imaging strategy to improve diagnostic accuracies (Midena et al., 2020; Tran and Pakzad-Vaezi, 2018; Acón and Wu, 2018; Garrity et al., 2018; Sandhu and Talks, 2005; Liu et al., 2020).

Thus, with the emergent concept of information fusion from different medical images, multimodal image-based DL algorithms have gained unique advantage of reflecting a more comprehensive understanding of the underlying pathology (Li et al., 2024; Jin et al., 2025; Wang et al., 2024; Yang et al., 2022). Currently, many studies have explored multimodal DL systems for diagnosing specific retinal disorders such as DR (Hervella et al., 2022), pathological myopia (Xu et al., 2025), AMD (Jin et al., 2022; Chen et al., 2021; De Silva et al., 2021; Yang et al., 2020), polypoidal choroidal vasculopathy (Xu et al., 2021), and glaucoma (Xiong et al., 2022), demonstrating advantages to single modality-based models to varying degrees. In contrast, using multimodal imaging-based DL models to simultaneously detect multiple fundus conditions remains poorly reported (Li et al., 2020; Sükei et al., 2024; Ma et al., 2025; Ou et al., 2024). Advances in this regard is exceptional meaningful, as current medical AI technologies are mainly aimed for situations like screening and auxiliary diagnosis, where comprehensive detection of the fundus is of vital importance.

In this multicenter study, we developed and evaluated DL models using both CFP and OCT to diagnose seven common retinal conditions, including normal retina and six pathologies: diabetic retinopathy (DR), dry and wet age-related macular degeneration (AMD), pathologic myopia (PM), epiretinal membrane (ERM), and macular edema (ME). This study aimed to evaluate the diagnostic performance and generalizability of bimodal DL models across different devices and scanning protocols. To further explore the capability of deep learning for precise and detailed disease grading, we conducted a study on the performance of ATN (atrophy, traction, and neovascularization) grading classification of PM.

## 2 Methods

This study was approved by the Institutional Review Board at Peking Union Medical College Hospital (PUMCH, approval number S-K2038), with written consent waived due to the retrospective nature of the study, and the de-identified data used. It was conducted in accordance with the tenets of the Declaration of Helsinki. The proposed workflow is illustrated in Supplementary Figure S1.

### 2.1 Datasets

#### 2.1.1 Inclusion/exclusion criteria

**FIGURE 1:**
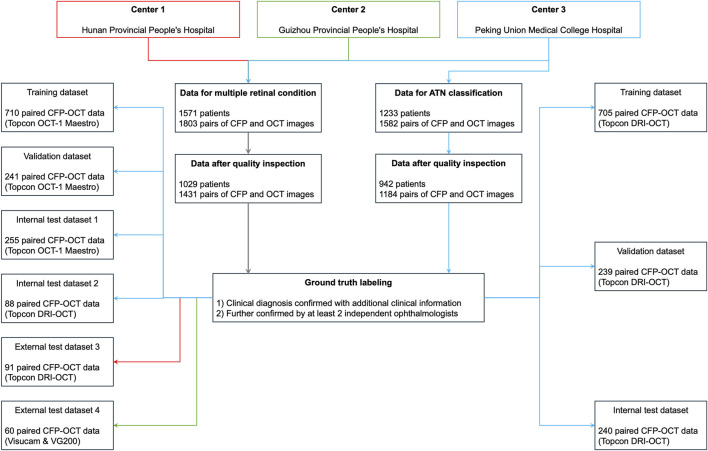
Datasets in the current study. ATN, A for atrophy, T for traction, N for neovascularization; CFP, color fundus photography; OCT, optical coherence tomography; PM, pathologic myopia.

We retrospectively collected the macula-centered retinal CFP and OCT images from 1 June 2018, to 1 June 2022. These images were obtained and diagnosed in PUMCH, Hunan Provincial People’s Hospital, and Guizhou Provincial People’s Hospital in China (Figure 1). Inclusion criteria: (1) Paired CFP and OCT images captured simultaneously using a single device or on the same day using separate devices. (2) For patients with multiple paired images, only those within an interval of more than 6 months were included. (3) Definite diagnosis of the ocular conditions which could be obtained from the medical history or corresponding imaging methods, including CFP, OCT, fluorescein angiography (FFA), and indocyanine green angiography (ICGA), etc. Exclusion criteria: Image quality judged not readable because of poor visibility or undesirable field of view, such as a small pupil and opacity of the refractive media.

#### 2.1.2 Image annotation

All images were de-identified and removed all personal information, including name, birth date, capture date, and gender, except for the diagnoses. We recruited seven licensed ophthalmologists from three hospitals to serve as readers (details are provided in Supplementary Table S1). Five readers labeled and checked the diagnosis by observing images and reviewing clinical information. Each image pair was evaluated by at least two readers, with two senior readers resolving controversies; unresolved cases were discarded. The quality of images was assessed before labeling, and images were excluded if they were judged not readable by even 1 reader.

#### 2.1.3 Dataset composition

We constructed multiple datasets from images acquired using various fundus photography (FP) and OCT devices for evaluation. We firstly collected CFP and OCT images captured with 3D OCT-1 Maestro (Topcon, Japan) consecutively in PUMCH. One CFP and 12 matching 9-mm radial-line cross-sectional spectral-domain (SD) OCT images centered on fovea were captured simultaneously by this device. This scanning pattern were used to create training dataset, validate dataset, and in-house test dataset 1 at the patient level, while ensuring the distribution manner of paired data was 8:1:1. The distribution of diagnosis was analyzed, and we found that the following retinal conditions were the most frequent diagnoses in this study, including normal condition, DR, dry AMD, wet AMD, PM, ERM, and ME. Thus, the 7 diagnoses with most clinical significance were selected to further test the diagnostic performance of the DL models while remaining simplicity.

To demonstrate the generalizability of our DL models, we further tested our DL models with three additional test datasets of images captured using different scanning patterns and devices from various hospitals. In test dataset 2 and 3, OCT and CFP images captured simultaneously were collected in PUMCH and Hunan Provincial People’s Hospital, respectively. These images were obtained using another swept-source (SS) OCT device, Topcon Deep Range Imaging (DRI) Triton OCT (Topcon, Japan). Of note, since patients visiting PUMCH were asked to adherence to the principle of using one imaging machine for follow-ups, ensuring no leakage of patient data between test dataset 1 and 2. In order to evaluate the feasibility of the DL models in a real-world clinical setting, the width of OCT scanning could be 6 mm, 9 mm, or 12 mm in test dataset 2. Moreover, considering that OCT and CFP images were usually not captured simultaneously using one device in clinical practice, we created another test dataset 4, in which OCT images were captured with a SS-OCT device (VG200, SVision Imaging, Ltd., China), and CFP images were captured with another camera (Zeiss Visucam 200, Zeiss, Germany) on the same day. Again, the width of OCT images in test dataset 4 could be ranging from 6 to 14 mm in various scanning patterns, including single line, radial lines, or cube scanning patterns. Considering that there was no published standard dataset of bimodal imaging for multiple retinal diseases, we did not use published datasets in this study.

To investigate the DL models’ capability for fine and detailed disease classification, we conducted a study on the ATN grading classification of PM using an independent ATN sub-dataset. The paired CFP and OCT images were captured using Topcon DRI OCT.

### 2.2 Development of the DL models

Considering that various retinal changes are evident on different imaging modalities, we propose a bimodal multi-instance learning network that targets OCT and CFP classification on seven retinal conditions to use information from two modalities fully based on previous work (Jin et al., 2022). Fundamentally, CFP and OCT imaging modalities capture distinct types of retinal information. CFP provides two-dimensional surface visualization, enabling the assessment of retinal structures and vascular patterns. In contrast, OCT generates cross-sectional views with depth resolution, revealing layer-specific architectural details. To address these inherent differences, our DL model incorporates modality-specific feature extraction branches. Specifically, CFP images are processed via spatial partitioning into patches to facilitate localized pathology detection, whereas OCT volumes are decomposed into individual B-scans for cross-sectional analysis.

**FIGURE 2:**
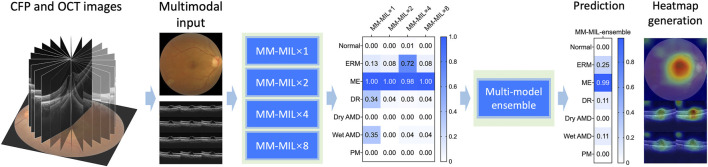
A simplified schematic workflow diagram of the deep learning system. We used stacked multimodal multi-instance learning (MM-MIL) models to predict retina conditions with color fundus photography (CFP) and optical coherence tomography (OCT) images.

In our framework, a pair of inputs contains a series of radial scanning OCT b-scans and a CFP image. We developed several models using multimodal multi-instance learning (MM-MIL) modules, and the model of best performance was selected for further analysis. The proposed MM-MIL framework employs adaptive attention weights that dynamically prioritize modality-specific features based on diagnostic relevance. For surface-level pathologies, the model emphasizes CFP-derived features. Conversely, for structural abnormalities like macular edema, it assigns greater weight to OCT-based features. This adaptive integration allows the model to effectively leverage the complementary strengths of each imaging modality, enhancing diagnostic accuracy. The AI framework describes in Supplementary Figure S2. A simplified schematic workflow is shown in Figure 2. We stacked 1, 2, 4, and 8 MM-MIL modules in the first four models respectively. As for the fifth model, we ensembled the outputs from four MM-MIL modules that worked independently to gain the final decision, which simulated the process in the real world that multiple physicians make the decision together (Wu et al., 2024). Finally, we developed five models for bimodal imaging: MM-MIL×1, ×2, ×4, ×8, and -ensemble. Through empirical evaluation, we found that the ×4 configuration achieved the best performance and selected it as our default architecture. The ensemble predictions are generated by this optimized MM-MIL×4 model, which processes the same multi-modal inputs through four MM-MIL modules. For visualization, we employed attention-weighted activation maps to highlight areas of interest in the model.

In order to compare the performance of models based on various imaging modalities, DL models for only CFP and for only OCT were developed. Since OCT scans consisting of a sequence of images are suitable for MILs, the MIL method was also applied to OCT images for classification. For CFP images, a classic and efficient deep learning algorithm, Resnet50, was trained to achieve multi-label classification. (He et al., 2016). Therefore, in our study, three DL models, CFP-MIL, OCT-MIL, and Fusion-MIL, were developed for CFP images, OCT images, and bimodal images, respectively.

### 2.3 Evaluation of the DL models and statistical analysis

The diagnostic performance of our DL models was evaluated using four independent test datasets for various purposes. (Yang et al., 2023). We first compared the five DL models based on bimodal imaging, and the models with the best performance were used for further evaluation. Areas under receiver operating characteristic curves (AUC) were computed for each diagnosis with a 95% confidence interval (CI). Some CFP and OCT images had more than one diagnosis label, so accuracy was also evaluated. We calculated “complete accuracy” to investigate whether all diagnoses given by DL models for one case were the same as ground truth, and “partial accuracy” to find out whether at least one of the diagnoses given by DL models for one case was the same as ground truth. According to the results from model selection, a one-sided test can be used. The DeLong test is used for comparing AUC values, whereas accuracy is compared with a chi-square test.

We also devised another experimental setting to evaluate the superiority of DL models based on bimodal imaging of CFP and OCT to models based on single-modal imaging. For each diagnosis, the diagnostic performance of DL models based on CFP, OCT, and bimodal imaging were evaluated and compared with each other. The evaluated retinal conditions were common in clinical settings and were not difficult to diagnose for most ophthalmologists. Therefore, the diagnostic performance of human ophthalmologists was not evaluated and compared with DL models.

## 3 Results

### 3.1 Demographics and datasets

For detecting multiple retinal conditions, 1803 pairs of CFP and OCT images from 1,571 patients meeting the inclusion criteria were initially reviewed, and we finally included 1445 CFP and OCT pairs from 1,029 patients after checking the image quality. Of these, 1,294 image pairs from PUMCH were allocated to the training, validation, test dataset 1, and test dataset 2. Ninety-one image pairs from Hunan Provincial People’s Hospital were used for testing dataset 3 and 60 images pairs from Guizhou Provincial People’s Hospital for testing dataset 4. The mean age (standard deviation, SD) in the three hospitals was 53.39 (19.44), 55.64 (10.53), and 57.45 (18.08) years, respectively, with no significant difference found among them. Table 1 summarizes demographic information and the distribution of the 7 retinal conditions in our in-house and external datasets. Sample images from the four testing datasets are shown in Supplementary Figure S3. Captured using various devices, these images differ in contrast, clarity, and choroidal penetration depth. For ATN classification of PM, the demographic and data feature are listed in Table 2.

**TABLE 1:** Patient demographics and dataset distribution for multiple retinal conditions across institutions.

Institution	Peking union medical college hospital				Hunan provincial People’s hospital	P value	Guizhou provincial People’s hospital	P value
Dataset (Device)	Training dataset (Maestro)	Validate dataset (Maestro)	Test dataset 1 (Maestro)	Test dataset 2 (DRI)	Test dataset 3 (DRI)		Test dataset 4 (Visucam VG200)	
No. of patients	911				70	-	48	-
No. of women, n (%)	536 (58.84)				36 (51.43)	0.226	23 (47.92)	0.133
Age, years (mean ± SD)	53.39 ± 19.44				55.64 ± 10.53	0.342	57.45 ± 18.08	0.162
No. of image pairs, n	710 (49.13)	241 (16.68)	255 (17.65)	88 (6.09)	91	-	60	-
No. of eye, n (%)	700 (48.92)	238 (16.63)	255 (17.82)	88 (6.15)	90	-	60	-
No. of eyes with normal condition, n (%)	358 (42.12)	117 (40.07)	126 (39.50)	0 (0.00)	15 (12.93)		11 (14.47)	
No. of eyes with ERM, n (%)	144 (16.94)	53 (18.15)	57 (17.87)	22 (20.18)	18 (15.52)		15 (19.74)	
No. of eyes with ME, n (%)	104 (12.24)	35 (11.99)	41 (12.85)	21 (19.27)	30 (25.86)		10 (13.16)	
No. of eyes with DR, n (%)	106 (12.47)	37 (12.67)	41 (12.85)	17 (15.60)	27 (23.28)		7 (9.21)	
No. of eyes with dry AMD, n (%)	64 (7.53)	21 (7.19)	26 (8.15)	14 (12.84)	10 (8.62)		14 (18.42)	
No. of eyes with wet AMD, n (%)	39 (4.59)	14 (4.79)	14 (4.39)	16 (14.68)	7 (6.03)		8 (10.53)	
No. of eyes with PM, n (%)	35 (4.12)	15 (5.14)	14 (4.39)	19 (17.43)	9 (7.76)		11 (14.47)	
P value (Test dataset 1 as reference)	0.993	0.997	-	<0.001*	<0.001*		<0.001*	

ERM, epiretinal membrane; ME, macular edema; DR, diabetic retinopathy; AMD, age-related macular degeneration; PM, pathologic myopia; SD, standard deviation; Maestro, 3D OCT-1, Maestro (Topcon, Japan); DRI, Deep Range Imaging Triton OCT (Topcon, Japan); VG200, VG200 OCT (SVision Imaging, China); Visucam, Zeiss Visucam 224 (Zeiss, Germany).

*indicates a *P* value <0.05 when comparing demographic characteristics (sex and age) or retinal condition distribution between two datasets.

**TABLE 2:** Patient demographics and dataset distribution for ATN classification in pathologic myopia.

Institution	Training dataset	Validation dataset	Test dataset
No. of eyes	705	705	705
Right	239	239	239
Left	240	240	240
No. of patients	354	354	354
Male	114	114	114
Female	126	126	126
Age, years (mean ± SD)	351	351	351
Atropic component			
A0	103	35	35
A1	35	61	56
A2	35	100	99
A3	173	34	43
A4	182	9	7
Tractional component			
T0	399	127	139
T1	127	63	58
T2	139	36	30
T3	665	7	7
T4	177	3	3
T5	63	3	3
Neovascular component			
N0	571	188	194
N1	188	5	6
N2a	194	11	10
N2s	953	35	30

ATN, A for atrophy, T for traction, and N for neovascularization; SD, standard deviation.

### 3.2 Model selection

We developed several MIL models and found that the MIL-Ensemble model had the best diagnostic performance on test dataset 1. The MIL-Ensemble model outperformed other MIL variants, achieving a sensitivity of 0.782 (95% CI: 0.726–0.838) and specificity of 0.967 (95% CI: 0.946–0.988). It achieved the highest AUC for detecting normal condition (AUC = 0.995, 95% CI 0.985–1.000), followed by PM (AUC = 0.985, 95% CI 0.941–1.000), wet AMD (AUC = 0.977, 95% CI 0.922–1.000), DR (AUC = 0.976, 95% CI 0.943–1.000), ME (AUC = 0.970, 95% CI 0.933–1.000), ERM (AUC = 0.927, 95% CI 0.879–0.974), and dry AMD (AUC = 0.847, 95% CI 0.752–0.942). The detailed results were shown in Supplementary Table S2. Thus, MIL-Ensemble model was selected for further evaluation.

### 3.3 Performance evaluation

Diagnostic performances of the selected MIL model for classifying retinal conditions based on CFP and OCT pairs (Fusion-MIL), CFP (CFP-MIL), and OCT (OCT-MIL) were evaluated and compared. In the test dataset 1, the CFP and OCT images were captured using Topcon OCT-1 Maestro, which was the same as the device used in the training and validation datasets. As detailed in Table 3, Fusion-MIL showed a higher overall AUC of 0.954 (95% CI 0.934–0.973) than CFP-MIL (AUC = 0.903, 95% CI 0.875–0.930, *P* < 0.001) and OCT-MIL (AUC = 0.928, 95% CI 0.904–0.952, *P* = 0.012). For each retinal condition, Fusion-MIL also had the best performance except in eyes with wet AMD, in which the AUC of Fusion-MIL (AUC = 0.977, 95% CI 0.922–1.000) was slightly lower than that of OCT-MIL (AUC = 0.978, 95% CI 0.925–1.000, *P* = 0.954), but still significantly higher than that of CFP-MIL (AUC = 0.903, 95% CI 0.795–1.000, *P* = 0.008) (Table 3). To demonstrate the ability of generalization of the MIL models, we further tested models with images captured using another device (Topcon DRI-OCT) with various scanning widths. Fusion-MIL also showed a higher overall AUC of 0.985 (95% CI 0.971–0.999) than CFP-MIL (AUC 0.876, 95% CI 0.841–0.910, *P* < 0.001) and OCT-MIL (AUC 0.982, 95% CI 0.966–0.998, *P* = 0.421) (Table 3). The diagnostic performance of Fusion-MIL on each retinal disease was superior to CFP-MIL and OCT-MIL, except for ERM and ME. The diagnostic performance of Fusion-MIL on ERM and ME (AUC = 0.951, 95% CI 0.887–1.000, and 0.989, 95% CI 0.956–1.000, respectively) was slightly lower than that of OCT-MIL (AUC = 0.972, 95% CI 0.923–1.000, *P* = 0.450, and 0.989, 95% CI 0.958–1.000, *P* = 0.987, respectively), but much higher than that of CFP-MIL (AUC = 0.700, 95% CI 0.568–0.832, *P* = 0.002, and 0.783, 95% CI 0.660–0.906, *P* < 0.001, respectively) (Table 3).

**TABLE 3:** AUC (95% CI) of Fusion-MIL (CFP + OCT), CFP-MIL, and OCT-MIL model for each retinal condition in 4 test datasets.

Fundus condition	Test dataset 1	*P* value	Test dataset 2	*P* value	Test dataset 3	*P* value	Test dataset 4	*P* value
	(Maestro)		(DRI)		(DRI)		(Visucam + VG200)	
Normal condition								
CFP + OCT	0.995 (0.985–1.00)	-	-	-	0.983 (0.929–1.000)	-	0.983 (0.970–0.996)	-
Only CFP	0.981 (0.964–0.998)	0.021*	-	-	0.978 (0.916–1.000)	0.785	0.958 (0.938–0.978)	0.042*
Only OCT	0.981 (0.963–0.998)	0.102	-	-	0.994 (0.963–1.000)	0.317	0.979 (0.965–0.993)	0.21
ERM								
CFP + OCT	0.927 (0.879–0.974)	-	0.951 (0.887–1.000)	-	0.951 (0.880–1.000)	-	0.846 (0.717–0.975)	-
Only CFP	0.821 (0.752–0.890)	<0.001*	0.700 (0.568–0.832)	0.002*	0.826 (0.704–0.948)	0.012*	0.676 (0.513–0.838)	0.038*
Only OCT	0.886 (0.828–0.944)	0.221	0.972 (0.923–1.000)	0.450	0.935 (0.854–1.000)	0.624	0.822 (0.685–0.958)	0.712
ME								
CFP + OCT	0.970 (0.933–1.000)	-	0.989 (0.956–1.000)	-	0.975 (0.936–1.000)	-	0.974 (0.904–1.000)	-
Only CFP	0.893 (0.827–0.959)	0.003*	0.783 (0.660–0.906)	<0.001*	0.840 (0.746–0.934)	0.001*	0.820 (0.655–0.985)	0.008*
Only OCT	0.969 (0.932–1.000)	0.956	0.989 (0.958–1.000)	0.987	0.955 (0.902–1.000)	0.210	0.984 (0.929–1.000)	0.841
DR								
CFP + OCT	0.976 (0.943–1.000)	-	0.999 (0.989–1.000)	-	0.973 (0.929–1.000)	-	0.997 (0.970–1.000)	-
Only CFP	0.963 (0.922–1.000)	0.412	0.991 (0.959–1.000)	0.125	0.966 (0.917–1.000)	0.745	0.995 (0.956–1.000)	0.876
Only OCT	0.923 (0.866–0.980)	0.018*	0.988 (0.952–1.000)	0.334	0.930 (0.862–0.999)	0.045*	0.952 (0.839–1.000)	0.012*
Dry AMD								
CFP + OCT	0.847 (0.752–0.942)	-	0.972 (0.911–1.000)	-	0.963 (0.880–1.000)	-	0.944 (0.859–1.000)	-
Only CFP	0.789 (0.683–0.895)	0.156	0.876 (0.755–0.996)	0.024*	0.898 (0.767–1.000)	0.089	0.932 (0.838–1.000)	0.712
Only OCT	0.809 (0.706–0.912)	0.432	0.957 (0.881–1.000)	0.621	0.946 (0.847–1.000)	0.534	0.893 (0.778–1.000)	0.205
Wet AMD								
CFP + OCT	0.977 (0.922–1.000)	-	0.997 (0.980–1.000)	-	1.000 (1.000–1.000)	-	0.998 (0.973–1.000)	-
Only CFP	0.903 (0.795–1.000)	0.008*	0.926 (0.836–1.000)	0.003*	0.922 (0.792–1.000)	0.042*	0.952 (0.847–1.000)	0.078
Only OCT	0.978 (0.925–1.000)	0.954	0.984 (0.939–1.000)	0.245	0.997 (0.970–1.000)	0.501	0.957 (0.857–1.000)	0.112
PM								
CFP + OCT	0.985 (0.941–1.000)	-	1.000 (1.000–1.000)	-	0.995 (0.961–1.000)	-	0.969 (0.895–1.000)	-
Only CFP	0.968 (0.904–1.000)	0.210	0.978 (0.931–1.000)	0.056	0.993 (0.955–1.000)	0.876	1.000 (1.000–1.000)	0.334
Only OCT	0.951 (0.872–1.000)	0.045*	1.000 (1.000–1.000)	0.501	0.999 (0.982–1.000)	0.712	0.985 (0.934–1.000)	0.621
Overall								
CFP + OCT	0.954 (0.934–0.973)	-	0.985 (0.971–0.999)	-	0.978 (0.958–0.997)	-	0.959 (0.931–0.987)	-
Only CFP	0.903 (0.875–0.930)	<0.001*	0.876 (0.841–0.910)	<0.001*	0.913 (0.876–0.951)	<0.001*	0.907 (0.867–0.947)	0.002*
Only OCT	0.928 (0.904–0.952)	0.012*	0.982 (0.966–0.998)	0.421	0.962 (0.938–0.986)	0.045*	0.941 (0.906–0.976)	0.089

AUC, areas under receiver operating characteristic curve; CFP, color fundus photography; CI, confidence interval; DR, diabetic retinopathy; AMD, dry age-related macular degeneration; ERM, epiretinal membrane; ME, macular edema; MIL, multiple instance learning; OCT, optical coherence tomography; PM, pathologic myopia; Maestro, 3D OCT-1, Maestro (Topcon, Japan); DRI, Deep Range Imaging Triton OCT (Topcon, Japan); Visucam, Zeiss Visucam 224 (Zeiss, Germany); VG200, VG200 OCT (SVision Imaging, China).

*P value <0.05 *P* value indicates the significance when comparing CFP-MIL, or OCT-MIL, with Fusion MIL (CFP + OCT), respectively.

To further test the generalization of Fusion-Net, we used an external test dataset that also included SS-OCT and CFP images (Topcon DRI-OCT). In the external test dataset 3, which used the same Topcon DRI-OCT as test dataset 2. Fusion-MIL (AUC = 0.978, 95% CI 0.958–0.997) (Table 2) presented better overall performance than CFP-MIL (AUC = 0.913, 95% CI 0.876–0.951, *P* < 0.001) and OCT-MIL (AUC = 0.962, 95% CI 0.938–0.986, *P* = 0.045), and was only slightly inferior to OCT-MIL in eyes with PM (Fusion-MIL AUC = 0.995, 95% CI 0.961–1.000 vs. OCT-MIL AUC = 0.999, 95% CI 0.982–1.000, *P* = 0.712) (Table 3).

Separate CFP and OCT examinations (Zeiss Visucam 224 and Svision VG200 SS-OCT) were performed in the external test dataset 4. Fusion-MIL (AUC = 0.959, 95% CI 0.931–0.987) still exhibited better overall performance than CFP-MIL (AUC = 0.907, 95% CI 0.867–0.947, *P* = 0.002) and OCT-MIL (AUC = 0.941, 95% CI 0.906–0.976, *P* = 0.089), but was slightly inferior to OCT-MIL in ME (Fusion-MIL AUC = 0.974, 95% CI 0.904–1.000 vs. OCT-MIL AUC = 0.984, 95% CI 0.929–1.000, *P* = 0.841) and PM (Fusion-MIL AUC = 0.969, 95% CI 0.895–1.000 vs. OCT-MIL AUC = 1.000, 95% CI 1.000–1.000, *P* = 0.621), and inferior to CFP-MIL in PM (Fusion-MIL AUC = 0.969, 95% CI 0.895–1.000 vs. CFP-MIL AUC = 1.000, 95% CI 1.000–1.000 *P* = 0.334) (Table 3).

### 3.4 Visualization and additional analyses

**FIGURE 3:**
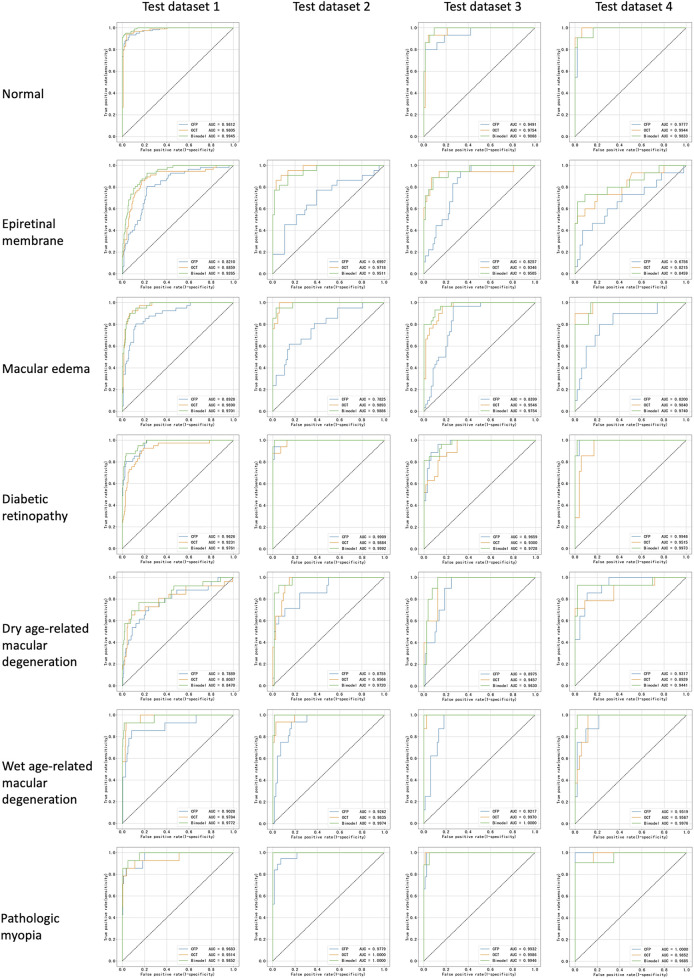
Receiver operating characteristic curves for all retinal conditions in test datasets. AUC, areas under receiver operating characteristic curve.

**FIGURE 4:**
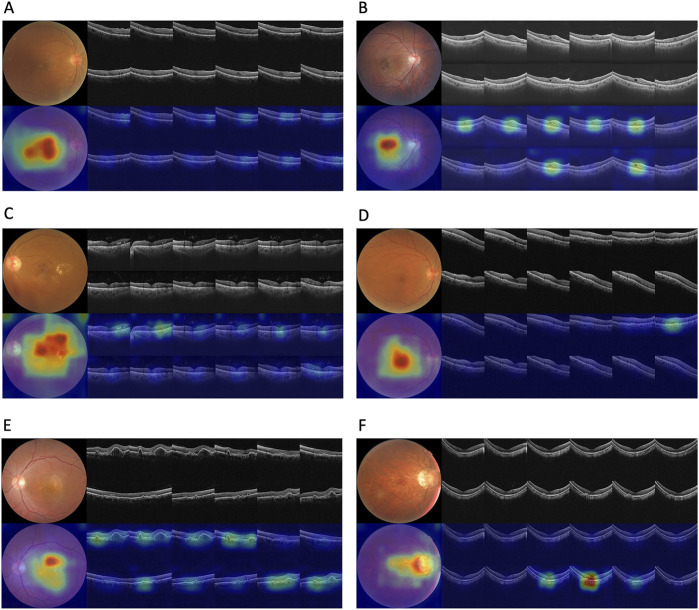
Original images and their activation heatmaps of abnormalities detected by deep learning models demonstrating representative changes of various retinal diseases. Original color fundus photography and optical coherence tomography images (above) and corresponding activation heatmaps (bellow) are shown.

Corresponding ROCs for the retinal conditions in the four test datasets are shown in Figure 3. The ROCs of Fusion-MIL were higher than those of CFP-MIL and OCT-MIL for most individual diagnosis. Figure 4 shows the examples of original CFP and OCT images and their activation heatmaps for each retinal condition generated by Fusion-MIL model. Abnormalities were obviously highlighted in various shapes, sizes, and locations according to each retinal condition. The confusion matrices for all condition are shown in Supplementary Figure S4, where it can accurately classify true-positive and true-negative cases for most retinal conditions, except for dAMD, ERM, and ME, for which the model exhibited comparatively higher false-negative rates.

Considering that some eyes suffered multiple diseases (for example, with both DR and ME), and their images had several labels of diagnoses, we further calculated the accuracy three MIL models across the four test datasets. The Fusion-MIL had higher complete accuracy (0.717–0.852) and partial accuracy (0.857–0.977) than CFP-MIL (complete accuracy 0.429–0.608, and partial accuracy 0.670–0.817) and OCT-MIL (complete accuracy 0.473–0.624, and partial accuracy 0.767–0.841) in all the four test datasets (Table 4). Of note, the difference in complete accuracy between Fusion-MIL and CFP/OCT-MIL models (difference up to 0.3) seemed to be more significant than that in partial accuracy (difference <0.15) (details in Supplementary Figures S5, 6).

**TABLE 4:** Complete accuracy and partial accuracy of MIL models in 4 test datasets.

Test dataset	Diagnostic accuracy	CFP + OCT	CFP	*P* value	OCT	*P* value
Test dataset 1	Complete accuracy	0.733	0.608	<0.01*	0.624	<0.01*
	Partial accuracy	0.898	0.804	<0.01*	0.828	<0.01*
Test dataset 2	Complete accuracy	0.852	0.443	<0.01*	0.580	<0.01*
	Partial accuracy	0.977	0.727	<0.01*	0.841	<0.01*
Test dataset 3	Complete accuracy	0.736	0.429	<0.01*	0.473	<0.01*
	Partial accuracy	0.857	0.670	<0.01*	0.824	<0.01*
Test dataset 4	Complete accuracy	0.717	0.483	<0.01*	0.500	<0.01*
	Partial accuracy	0.900	0.817	<0.01*	0.767	<0.01*

CFP, color fundus photography; OCT, optical coherence tomography; MIL, multiple instance learning.

Complete accuracy, defined as all diagnoses given by MIL, models for one case are the same as ground truth.

Partial accuracy, defined as at least one of the diagnoses given by MIL, models for one case is the same as ground truth, while no misdiagnosis is given.

*P* value indicates the significance when comparing CFP-MIL, or OCT-MIL, with Fusion MIL (CFP + OCT), respectively.

*P value <0.05.

Additionally, we evaluated the model’s capability to classify pathologic myopia using the ATN classification system, which integrates both CFP and OCT imaging modalities, thereby further demonstrating the clinical applicability of our approach. The Fusion-MIL model achieved AUC values ranging from 0.902 to 0.997 for the atrophic component, 0.869 to 0.957 for the tractional component, and 0.742 to 0.976 for the neovascular component (detailed results are presented in Table 5).

**TABLE 5:** AUC of Fusion-MIL for ATN classification of PM and therapy decision.

Atrophic component	AUC (95% CI)	Tractional component	AUC (95% CI)	Neovascular component	AUC (95% CI)
A0: no myopic retinal lesions	0.997 (0.975–1.000)	T0: no macular schisis	0.957 (0.850–1.000)	N0: no myopic CNV	0.898 (0.861–0.935)
A1: tessellated fundus only	0.914 (0.775–1.000)	T1: inner or outer foveoschisis	0.878 (0.780–0.940)	N1: macular lacquer cracks	0.742 (0.572–0.912)
A2: diffuse chorioretinal atrophy	0.902 (0.757–0.964)	T2: Inner + outer foveoschisis	0.887 (0.780–0.950)	N2a: active CNV	0.976 (0.833–1.000)
A3: patchy chorioretinal atrophy	0.918 (0.625–1.000)	T3: foveal detachment	0.953 (0.625–1.000)	N2s: scar/Fuchs spot	0.904 (0.815–0.993)
A4: complete macular atrophy	0.965 (0.800–1.000)	T4: full-thickness MH	0.869 (0.780–0.995)		
		T5: MH + retinal detachment	0.982 (0.875–1.000)		

ATN, A for atrophy, T for traction, and N for neovascularization; AUC, areas under receiver operating characteristic curve; CI, confidence interval; CNV, choroidal neovascularization; MH, macular hole; PM, pathologic myopia; VEGF, vascular endothelial growth factor.

The learning curves of the Fusion-MIL model were analyzed to assess training stability and convergence. Supplementary Figure S7 illustrates the progression of the mean AUC, mean average precision (AP), and loss metrics over training epochs. The mean AUC and AP steadily improved as training progressed, demonstrating enhanced model discrimination and precision. Concurrently, the learning curves demonstrate a general decline in both training and validation losses, indicating model convergence, while the validation loss remains relatively lower than the training loss throughout the epochs.

## 4 Discussion

In this study, we introduced a DL model based on the MM-MIL algorithm for classifying multiple retinal conditions using CFP and OCT images. The Fusion-MIL based on bimodal imaging showed reliable performance for 7 common retinal conditions, including normal fundus, ERM, ME, DR, dry AMD, wet AMD, and PM. It achieved stable AUC values of 0.954–0.985 across four different test datasets. Fusion-MIL also outperformed CFP-MIL and OCT-MIL models which were based on single-modal images. The ATN classification of PM demonstrated that Fusion-MIL could also be used for more detailed classification and treatment decision.

The proposed bimodal imaging strategy using CFP and OCT emulates real-world clinical evaluation processes. Our results show that this hybrid method combines analysis of en-face CFP and cross-sectional OCT images, which can obtain more information from medical data and improve the performance for various retinal conditions (Jaffe and Caprioli, 2004). Kang et al. reported a multimodal imaging-based DL model for five retinal vascular diseases, including diabetic macular edema (DME), neovascular AMD, myopic choroidal neovascularization (mCNV), and branch and central retinal vein occlusion (BRVO/CRVO) using images of CFP, OCT, and fluorescein angiography (FA), with or without indocyanine green angiography (ICGA) (Kang et al., 2021). Their models were trained with images from 2,992 eyes and the AUC detecting wet AMD (one condition also detected by our model) achieved 0.990. In our study, the AUCs detecting wet AMD in the four test datasets were 0.9772, 0.9974, 1.0000, and 0.9976, respectively, similar to Kang’s study. Therefore, more imaging modalities (e.g., invasive methods like FA and ICGA) in addition to CFP and OCT might not improve diagnostic performance significantly. Li et al. also trained a CFP- and FA-based bimodal DL model for detecting AMD and PM with two public datasets (Ichallenge-AMD and Ichallenge-PM). It showed AUC values of 0.756 for AMD and 0.986 for PM, respectively, (Li et al., 2020), which is not superior to our Fusion-MIL, as it’s AUCs for dry AMD, wet AMD, and PM were 0.847–0.972, 0.977–1.000, and 0.969-1.000 across four test datasets, respectively. Therefore, bimodal imaging of CFP and OCT rather than other imaging methods might be more likely to improve DL model’s diagnostic performance, which is concordant to the clinical practice that CFP and OCT are the most regular non-noninvasive examinations for retinal diseases (Li et al., 2018). Perhaps benefiting from the efficient diagnostic information provided by bimodal imaging, our MIL models were trained on a relatively small dataset (710 image pairs) and obtained a robust performance on the test datasets (mean AUC above 0.95). Furthermore, DL models relying on invasive examinations (e.g., FA and ICGA) are unsuitable for screening purposes. In contrast, as CFP and OCT are widely used, non-invasive, rapid, convenient, and repeatable examinations, our method may have greater potential to be applied to a broader range of diseases.

However, we noticed that Fusion-Net was inferior to OCT-Net or CFP-Net in detecting some specific diseases occasionally. For example, Fusion-Net (AUC 0.9685, 95% CI 0.8951–1.0000) was inferior to CFP-Net (AUC 1.0000, 95% CI 1.0000–1.0000) in detecting PM when using test dataset 4. As PM usually presents typical tessellated fundus in CFP images, but OCT features that showed no apparent abnormalities might decrease the diagnostic performance of Fusion-Net for PM. In contrast, other retinal diseases, such as ERM, ME, and wet AMD, usually exhibit significant OCT lesions easily detected by Fusion-Net. Therefore, optimizing the weights of imaging features of different modalities for various diseases could further enhance DL models based on multimodal imaging.

For a diagnostic model, its performance metrics usually shows a decrease trend from validation to test to external test sets to indicate the model is not overfitting. However, in our study, AUC values in test dataset 2 – 4 were even higher than that in test dataset 1, which had a more similar dataset composition with training and validation sets. Several reason may be considered: First, differences in imaging devices and their inherent technical specifications likely contributed to performance disparities. Test dataset 1 comprised images acquired using the 3D OCT-1 Maestro (Topcon, Japan), a SD-OCT system, which was also employed for model training and internal validation. In contrast, test datasets 2 and 3 were derived from the DRI Triton OCT (Topcon, Japan), and test dataset 4 from the VG200 (SVision Imaging, China) - both SS-OCT systems. SS-OCT typically offers superior image clarity, resolution, and signal-to-noise ratio compared to SD-OCT, which may have enhanced the model’s ability to discern discriminative features, leading to improved diagnostic performance. Second, variations in dataset composition may have further influenced model performance. Although test datasets 2 and 3 were obtained using the same DRI Triton OCT device, their retinal condition distributions differed substantially, as test dataset 2 does not include “Normal” cases, resulting in a more balanced and simplified classification task. This structural difference may explain the higher AUC in test dataset 2 compared to test dataset 3. These findings underscore the importance of considering both imaging modality differences and dataset heterogeneity when evaluating AI model generalizability across diverse clinical settings.

In the current study, deep learning algorithms had the best diagnostic performance in ocular images with multiple disease labels. In the real-world clinical setting, comprehensive diagnoses are correlated with the urgency of referral and the necessity of treatment. For example, ME in eyes with wet AMD or DR could be an indication for treatment. In the current study, the Fusion-Net had the highest complete accuracy and partial accuracy than CFP-Net and OCT-Net and showed apparent advantages in giving disease labels on the whole. Our results suggest that bimodal imaging enhanced the DL models’ ability to minimize missed diagnoses by obtaining more diagnostic information. The Fusion-Net can reduce missed diagnoses, which is needed in scenarios of clinic diagnosis and community screening, and might assist doctors without abundant clinical experience in the early learning stage and clinical practice (Resnikoff et al., 2020).

A critical feature of this study is the test of generalization using bimodal imaging data from different devices from various manufacturers. Fauw et al. reported that a well-trained SD-OCT-based model for detecting retinal diseases was tested with SD-OCT images captured with a new scanning device; the performance was unsatisfying unless the models were retrained with images of new devices (De Fauw et al., 2018). Limited generalizability is also one of the common issues of DL models mentioned in many other studies (Wang et al., 2024; Goutam et al., 2022). However, in the current study, the SD-OCT and CFP-trained Fusion-Net models were tested with SS-OCT and CFP images captured with various devices, and diagnostic performance using the test dataset 2-4 remained stable. We speculated that one possible explanation is that Fusion-Net did not depend on a solitary imaging method, and the two imaging methods provided complementary information. Therefore, bimodal imaging improved the models’ adaptability. Another possible explanation is that our models extracted features automatedly rather than learned features by annotated lesions. These models avoid over-reliance on the characteristics of localized lesions. Once the style features of images changed, our models exhibited good identification ability.

In terms of the model’s visualization, the interpretability component leverages attention mechanisms to produce clinically meaningful visualizations, enhancing model transparency and aiding clinicians in understanding diagnostic decisions. Through multi-model ensembling, it generates attention-weighted heatmaps: OCT B-scans are ranked by attention scores to emphasize diagnostically significant cross-sections, while CFP images receive spatial overlays where intensity gradients highlight areas of focus. Although this enhances clinical utility, heatmaps may not accurately mirror underlying decision logic and may be susceptible to misinterpretation, reflecting the broader challenges of post-hoc explainability in medical imaging AI (Ennab and McHeick, 2024). There is also a risk of bias if training datasets lack demographic diversity, potentially undermining performance for underrepresented patient groups (Houssein et al., 2025; Nouis et al., 2025). Future work should explore hybrid or self-explainable AI approaches that embed interpretability into the model’s architecture, address bias via representative data and auditing, and rigorously validate outputs in real-world clinical settings—all supported by human oversight to ensure trust, accountability, and ethical deployment in healthcare (Nouis et al., 2025; Alkhanbouli et al., 2025; Gong et al., 2024).

There are several limitations in the current study. First, the Fusion-MIL model was not tested with published external dataset, because there are no such datasets of bimodal imaging for multiple retinal diseases currently, and we chose to verify DL methods with data from various centers. Even though, true external data collected independently is crucial for more convinced assessment of our model’s generalizability and potential of clinical translation. More importantly, closely following the latest regulatory guidance on AI/machine learning in medical devices, such as Good Machine Learning Practice (GMLP) for Medical Device Development, (FDA, 2021), provides a robust framework for model test and cross-model comparison. Thus, the comparison proposed in our study need to be interpreted with caution. Second, we did not compare the diagnostic performance of DL models with human ophthalmologists, because the tested retinal conditions were selected according to the prevalence in real-world clinical setting. Therefore, these conditions were not difficult for most ophthalmologists to diagnose, and this study focused on the comparison of DL-assisted diagnosis based on bimodal and single-model imaging, rather than comparison of DL methods and human. Further research is required to overcome these limitations. Third, the image data is relatively limited compared with other studies. Though the MIL models already achieved high classification accuracy, it remains unclear whether larger training dataset could help further improve model performance. Fourth, we included only 7 retinal conditions in this preliminary study. More retinal diseases, such as retinal vein occlusion, will be included in future investigation.

In conclusion, the Fusion-MIL model, based on bimodal imaging with CFP and OCT, achieved both accurate and comprehensive diagnoses of several retinal diseases. It outperformed models based on single-modal imaging and demonstrated non-inferior performance compared to other state-of-the-art multimodal models. The model’s stable performance across test datasets from different devices and medical centers suggests that it is highly generalizable to heterogeneous clinical settings. The simple combination of CFP and OCT, rather than more complex or invasive imaging methods, may be sufficient for automatic and effective detection of common retinal diseases. Future work could focus on optimizing the weighting of each imaging modality for different retinal diseases, expanding the size of training and testing datasets, evaluating the model with truly independent external datasets, and including human-machine comparisons. We believe that, with further validation across more retinal diseases, our approach has the potential to support fundus screening and early diagnosis of vision-threatening conditions in areas lacking access to experienced ophthalmologists.

## Data Availability

The original contributions presented in the study are included in the article/Supplementary Material, further inquiries can be directed to the corresponding authors.
