# Correction: Diagnostic performance and generalizability of deep learning for multiple retinal diseases using bimodal imaging of fundus photography and optical coherence tomography

**DOI:** 10.3389/fcell.2026.1813162

**Published:** 2026-03-12

**Authors:** Xingwang Gu, Yang Zhou, Jianchun Zhao, Hongzhe Zhang, Xinlei Pan, Bing Li, Bilei Zhang, Yuelin Wang, Song Xia, Hailan Lin, Jie Wang, Dayong Ding, Xirong Li, Shan Wu, Jingyuan Yang, Youxin Chen

**Affiliations:** 1 Department of Ophthalmology, Peking Union Medical College Hospital, Chinese Academy of Medical Sciences, Beijing, China; 2 Key Laboratory of Ocular Fundus Diseases, Chinese Academy of Medical Sciences, Beijing, China; 3 Vistel AI Lab, Visionary Intelligence Ltd., Beijing, China; 4 Department of Nuclear Medicine, Peking Union Medical College Hospital, Chinese Academy of Medical Sciences, Beijing, China; 5 Department of Ophthalmology, The First Affiliated Hospital of Zhejiang University, Hangzhou, Zhejiang, China; 6 Department of Ophthalmology, Hunan Provincial People’s Hospital, Changsha, China; 7 Department of Ophthalmology, Peking University Third Hospital, Beijing, China; 8 Department of Ophthalmology, Guizhou Provincial People’s Hospital, Guiyang, China; 9 Key Lab of DEKE, Renmin University of China, Beijing, China; 10 Beijing Hospital, National Center of Gerontology, Institute of Geriatric Medicine, Chinese Academy of Medical Sciences, Beijing, China

**Keywords:** deep learning, diagnosis, fundus photography, optical coherence tomography, retinal disease

An incorrect number was provided for the CAMS Initiative for Innovative Medicine. The correct number is (2023-12M-C&T-B-004, 2018-I2M-AI-001). The funder the National High Level Hospital Clinical Research Funding (2025-PUMCH-A-189) was erroneously omitted. The correct **funding** statement reads:

The author(s) declare that financial support was received for the research and/or publication of this article. Supported by the National Natural Science Foundation of China (82301239, 82200730, and 82271112), the National High Level Hospital Clinical Research Funding (2025-PUMCH-A-189), the CAMS Initiative for Innovative Medicine (2023-12M-C&T-B-004, 2018-I2M-AI-001), and Pharmaceutical collaborative innovation research project of Beijing Science and Technology Commission (Z191100007719002). Xinjiang Production and Construction Corps Science and Technology Project, Science and Technology Development Program in Major Fields (2022AB021). The sponsors or funding organizations had no role in the design or conduct of this research. The authors alone are responsible for the content and writing of the article.

The original article has been updated.

