# Supplementary material for: Diagnostic performance and generalizability of deep learning for multiple retinal diseases using bimodal imaging of fundus photography and optical coherence tomography
Source: Front Cell Dev Biol. 2025 Sep 11;13:1665173. doi: 10.3389/fcell.2025.1665173 (PMC12460420; doi:10.3389/fcell.2025.1665173)
Supplement: Supplementary file 1 [file DataSheet1.pdf]

Table of contents

Supplementary Figure 1. Workflow diagram of the current study.

Supplementary Figure 2. The schematic architecture of multimodal multi-instance learning (MM-MIL) based on combinations of color fundus photography (CFP) and optical coherence tomography (OCT) images.

Supplementary Figure 3. Sample images from four test datasets.

Supplementary Figure 4. Confusion matrices of Fusion-MIL for seven retinal conditions across four test datasets.

Supplementary Figure 5. Scatter plots of erroneous predictions by Fusion-MIL (A), CFP-MIL (B), and OCT-MIL (C).

Supplementary Figure 6. For images with multiple labels of diseases, the predictive correctness of each label using different imaging modalities.

Supplementary Figure 7. Learning curves for mean AUC (A), mean average precision (B), and loss (C).

Supplementary Table 1. The experience and position of the seven readers.

Supplementary Table 2. The diagnostic performance (95% CI) of various MIL methods for overall cases and each retinal condition on test dataset 1.

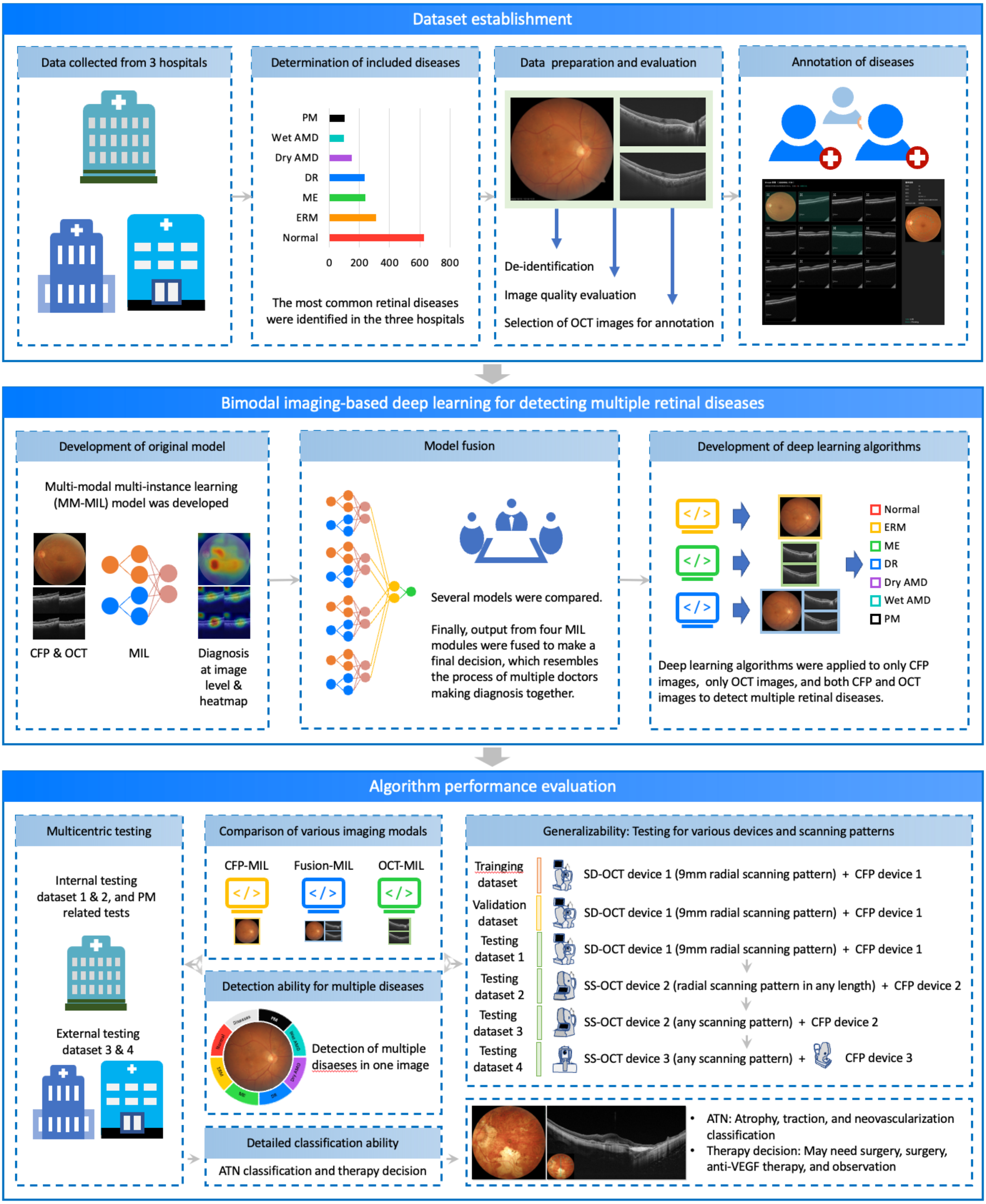

Supplementary Figure 1. Workflow diagram of the current study. Paired images were collected from three hospitals. After preprocessing the images, certified ophthalmologists annotated the images. Various deep learning algorithms were tested, and the one with the best performance which simulated decision making by

a group of experts was selected. The deep learning algorithm was trained to detect multiple retinal conditions, to grade ATN classification and to make therapy decisions for pathologic myopia. The performance of diagnosis, generalizability, and therapy decision was tested, and the performance based on various imaging modalities was also compared.

A.

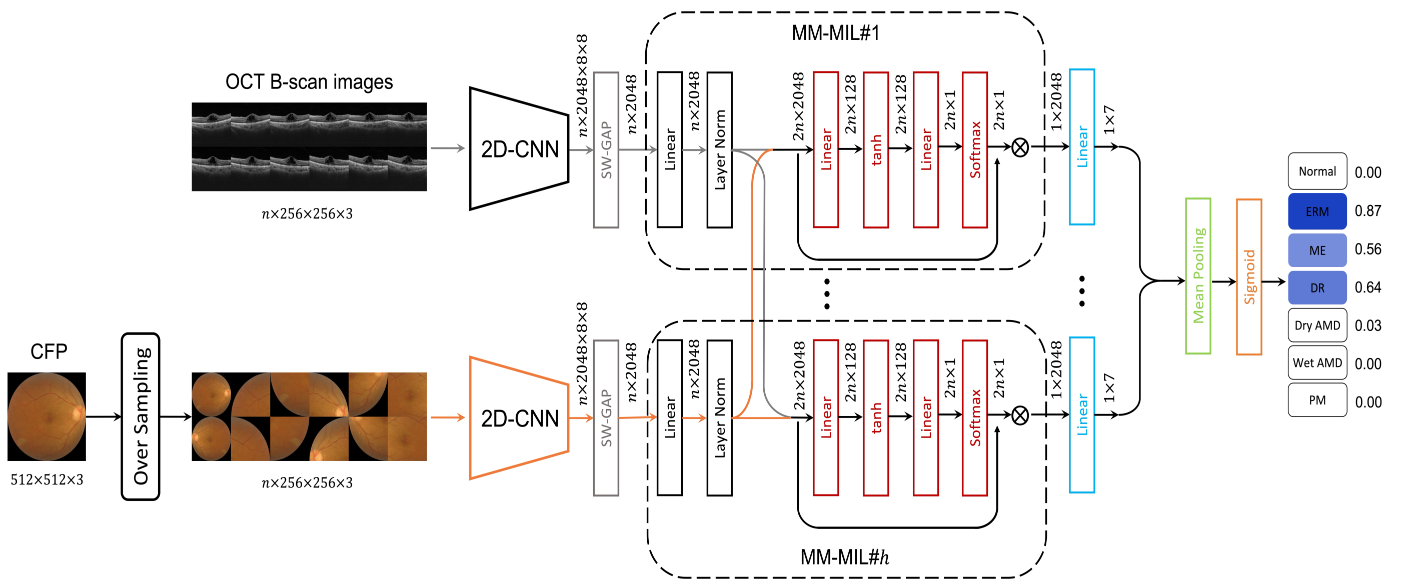

B.

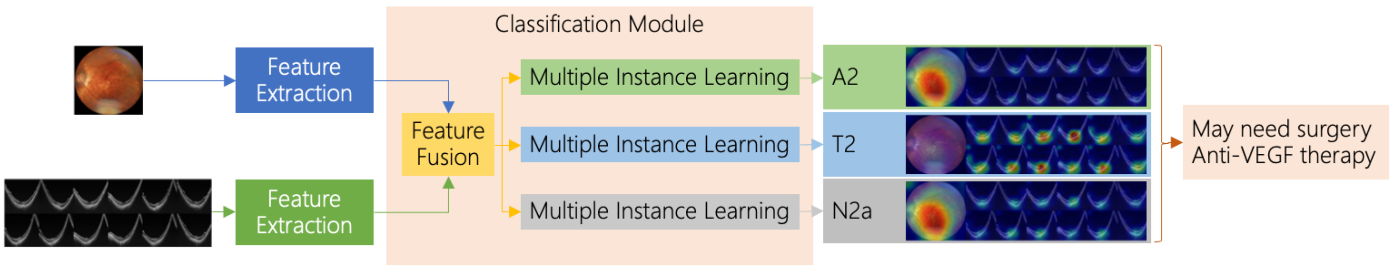

Supplementary Figure 2. A. The schematic architecture of multimodal multi-instance learning (MM-MIL) based on combinations of color fundus photography (CFP) and optical coherence tomography (OCT) images. For a given pair of inputs, we applied pseudo-oversampling to the CFP to generate a series of augmented CFPs. CFP and OCT images are then fed into a two-dimensional convolutional neural network (2D-CNN) to extract features, respectively. After spatial-wise global average pooling, the features from two modalities competed in the MM-MIL modules to obtain the contribution weights to the final diagnosis. MM-MIL module can stack multiple depending on the number of classes. Finally, the classifier predicted diagnosis probabilities.

The MM-MIL module contains three identical parts: (1) CFP pseudo-oversampling and feature extraction, (2) bimodal multi-instance feature fusion, and (3) result ensemble and classification. In order to balance the number of pictures for two modalities and entirely use information from two modalities, we applied a pseudo-oversampling on CFP. In more detail, we cropped the four corners and the center into five images and resized

them to the original image size. Then we flip the original image and these five images horizontally. Finally, we got 12 images with the same number of OCT modality. Due to the significant differences between CFP and OCT, we used two 2D-CNN networks to extract features, respectively, for they vary significantly. Employing spatial-wise global average pooling (SW-GAP) on the 2D-CNN features, we obtained the features of OCT and CFP.

MM-MIL, the second part, receives features of OCT and CFP and fuses them into one integrated feature. To make features from different modalities comparable, we proposed a cross-modal projection block, including a linear layer and a layer normalization. To make features compete with each other, we introduce an instance attention block including linear, tanh, linear, softmax layers in order. In this block, we computed instance weights for each feature and weighted sum them into an integrated feature.

According to the result in the second part, each MM-MIL head can compute the probability of each disease by employing a linear layer. Then, we ensembled those heads by a mean pooling and a sigmoid activation which was the prediction of an MM-MIL module.

B. A simplified schematic architecture of MIL for ATN (A for atrophy, T for traction, and N for neovascularization) classification of pathologic myopia and therapy decision making.

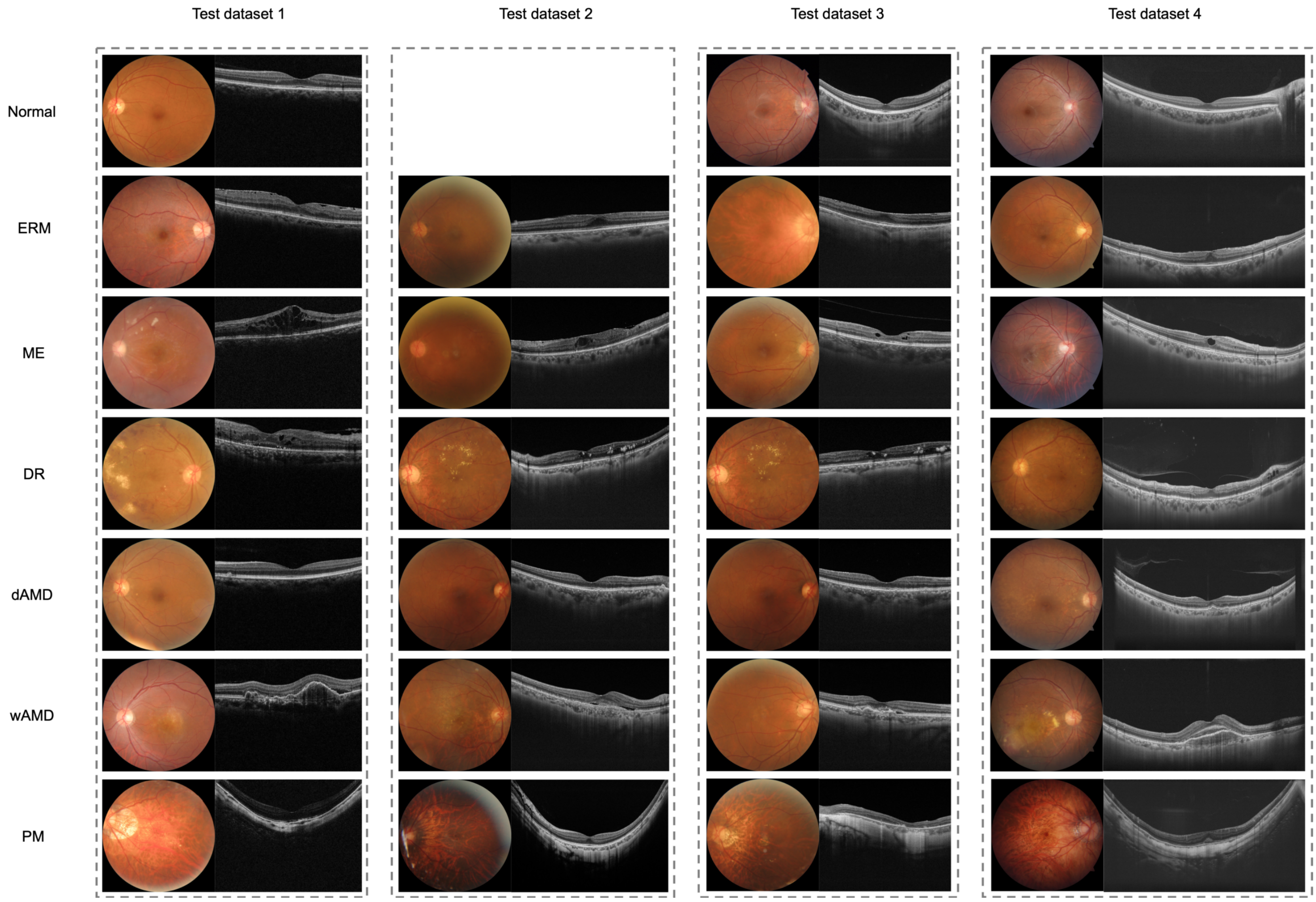

Supplementary Figure 3. Sample images from four test datasets. Images in test dataset 1 were captured using the Topcon 3D-OCT1 Maestro (CFP + 9 mm radial OCT). Images in test datasets 2 and 3 were captured using the Topcon DRI Triton (CFP + 6, 9, and 12 mm radial OCT; dataset 2 includes 6, 9, and 12 mm OCT images, whereas dataset 3 includes only 9 mm OCT images). Images in test dataset 4 were captured using the Zeiss VisuCam 200 (CFP) and VG200, SVision (OCT with various scanning patterns). These images differ in contrast, clarity, and choroidal penetration depth.

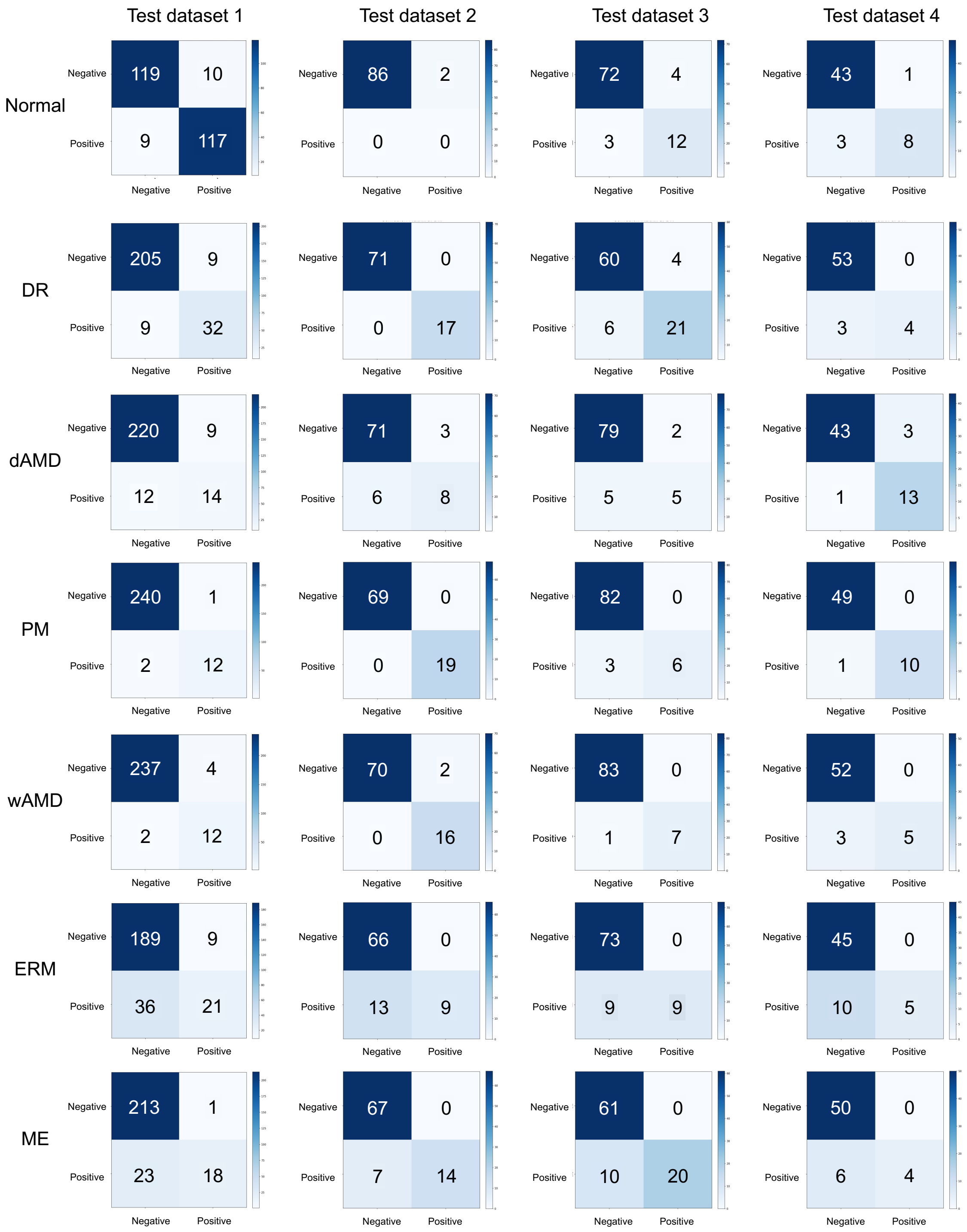

Supplementary Figure 4. Confusion matrices of Fusion-MIL for seven retinal conditions across four test datasets. The model accurately classifies true-positive and true-negative cases for most retinal conditions, except for dAMD, ERM, and ME, for which the model exhibited relatively higher false-negative rates.

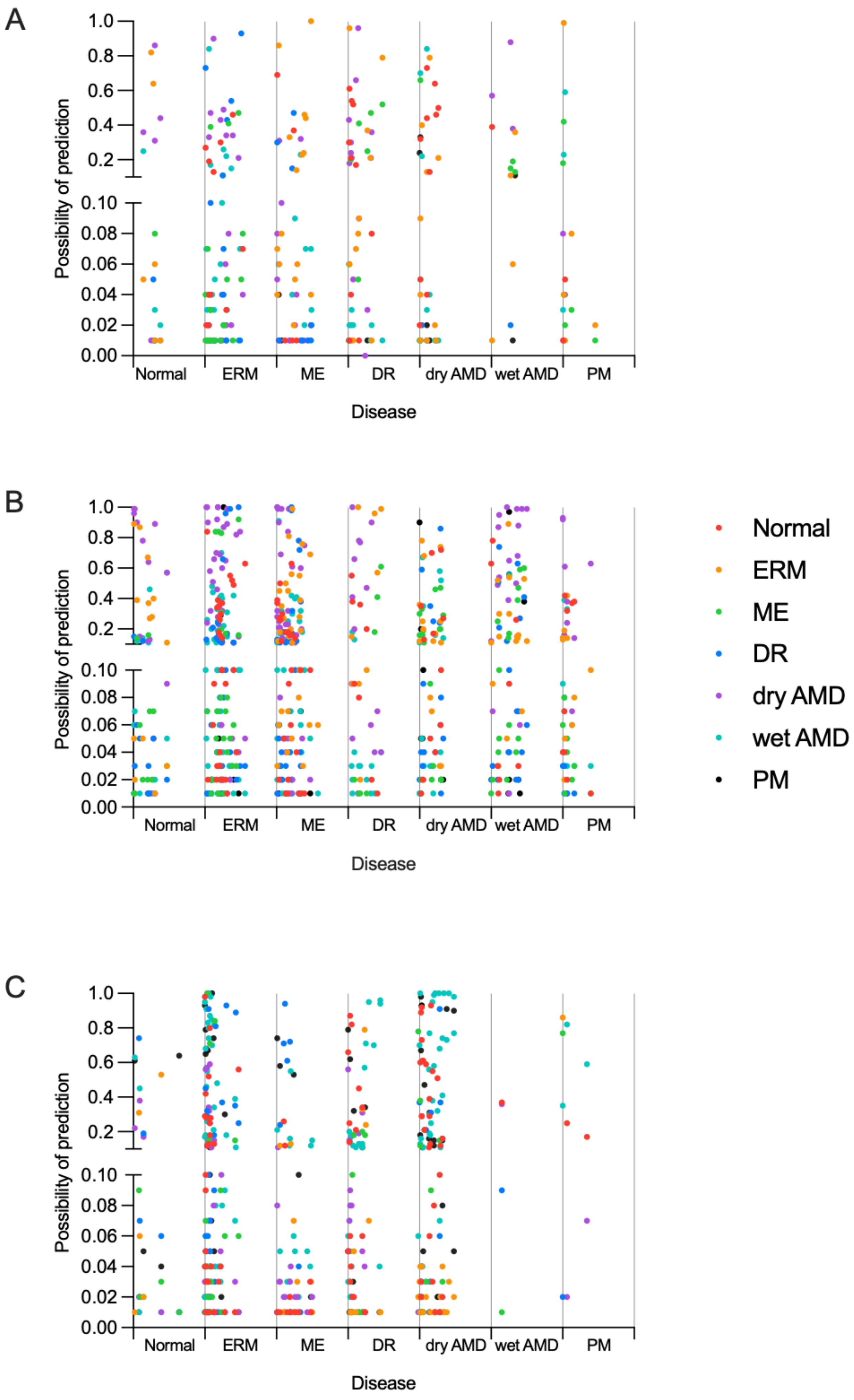
A
B
C
Possibility of prediction
1.0
0.8
0.6
0.4
0.2
0.10
0.08
0.06
0.04
0.02
0.00
Normal
ERM
ME
DR
dry AMD
wet AMD
PM
Disease
Normal
ERM
ME
DR
dry AMD
wet AMD
PM

Supplementary Figure 5. Scatter plots of erroneous predictions by Fusion-MIL (A), CFP-MIL (B), and OCT-MIL (C). The x-axis represents the predictive possibility of ground truth diagnosis by the deep learning algorithms, and the possibility of each retinal condition ranging from 0 to 1 in the corresponding regions from left to right. The y-axis represents the predictive possibility of erroneous diagnosis by the deep learning algorithms. Each dot represents an erroneous prediction. The number of erroneous dots in (A) is much fewer than those in (B) and (C). Dots of erroneous prediction based on CFP (B) mainly locate in the regions of ERM, ME, dry AMD, and wet AMD, which could be better recognized on OCT images. Dots of erroneous prediction based on OCT (C) mainly locate in the regions of ERM and dry AMD, which might have subtle changes in OCT images. On the contrary, dots of erroneous prediction based on bimodal imaging (A) in the regions of ERM and dry AMD are less than those based on CFP (B) and OCT (C).

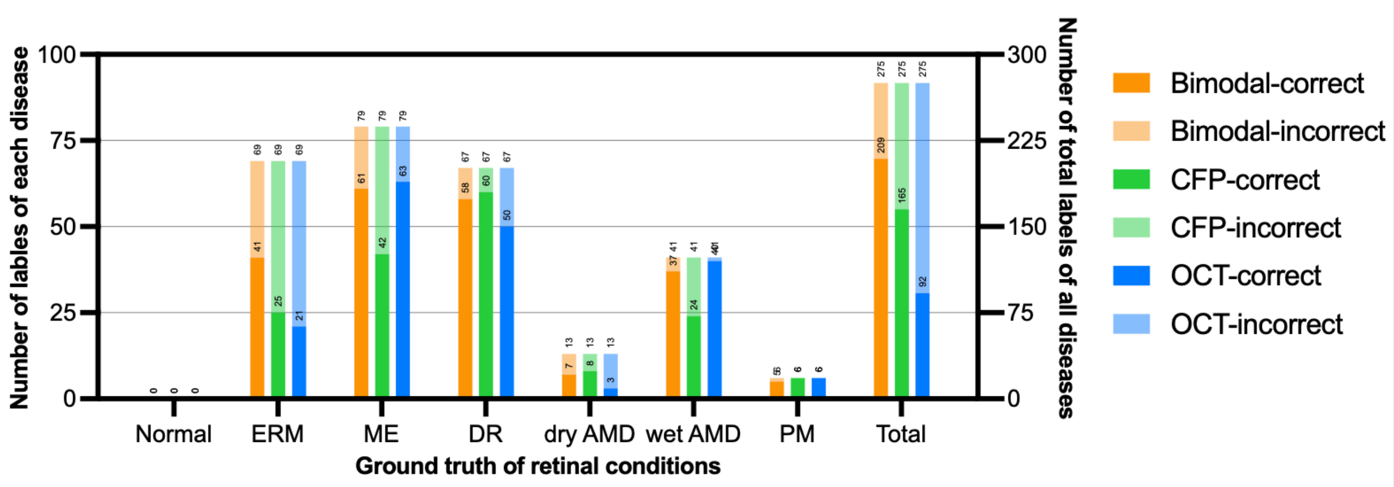

Supplementary Figure 6. For images with multiple labels of diseases, the predictive correctness of each label using different imaging modalities. The bimodal imaging-based deep learning algorithm had the best overall correctness, and its correctness on each disease is also shown.

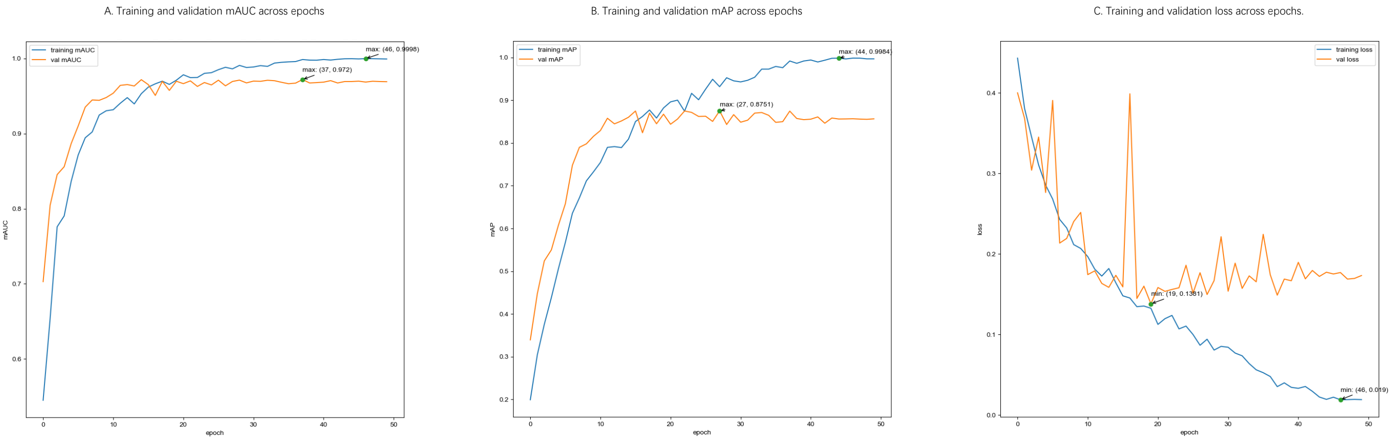

Supplementary Figure 7. Learning curves for mean AUC (A), mean average precision (B), and loss (C). The curves illustrate convergence trends and performance stability, with close alignment between training and validation metrics indicating effective generalization.

Supplementary Table 1. The experience and position of the seven readers.

Reader	Position	Years of Experience
1	Professor in retina	> 30
2	Professor in retina	> 20
3	Associate professor in retina	10
4	National certified attending in retina	9
5	National certified attending in retina	9
6	National certified attending in retina	8
7	Resident	4

Supplementary Table 2. The diagnostic performance (95% CI) of various MIL methods for overall cases and each retinal condition on test dataset 1.

		MIL models				
		MIL-1	MIL-2	MIL-4	MIL-8	MIL-ensemble
Overall performance	Sensitivity	0.7527 (0.6971-0.8084)	0.7734 (0.7162-0.8307)	0.7485 (0.6919-0.8052)	0.7570 (0.7000-0.8140)	0.7820 (0.7260-0.8380)
	Specificity	0.9597 (0.9347-0.9847)	0.9597 (0.9381-0.9813)	0.9586 (0.9351-0.9821)	0.9666 (0.9467-0.9864)	0.9669 (0.9459-0.9878)
	AUC	0.9261 (0.9030-0.9493)	0.9424 (0.9201-0.9648)	0.9545 (0.9352-0.9739)	0.9505 (0.9298-0.9713)	0.9538 (0.9342-0.9734)
	F1 score	0.8330 (0.7849-0.8810)	0.8483 (0.7995-0.8970)	0.8314 (0.7822-0.8806)	0.8425 (0.7946-0.8904)	0.8604 (0.8138-0.9070)
Retinal conditions	Normal	0.9895 (0.9768-1.0000)	0.9929 (0.9825-1.0000)	0.9937 (0.9838-1.0000)	0.9922 (0.9813-1.0000)	0.9945 (0.9854-1.0000)
	ERM	0.8871 (0.8294-0.9448)	0.9303 (0.8836-0.9769)	0.9198 (0.8701-0.9695)	0.9297 (0.8829-0.9766)	0.9265 (0.8787-0.9743)
	ME	0.8955 (0.8301-0.9609)	0.9404 (0.8894-0.9914)	0.9769 (0.9443-1.0000)	0.9606 (0.9186-1.0000)	0.9701 (0.9334-1.0000)
	DR	0.9647 (0.9248-1.0000)	0.9664 (0.9274-1.0000)	0.9683 (0.9305-1.0000)	0.9782 (0.9467-1.0000)	0.9761 (0.9430-1.0000)
	Dry AMD	0.7885 (0.6824-0.8947)	0.8332 (0.7351-0.9313)	0.8586 (0.7663-0.9508)	0.8455 (0.7501-0.9409)	0.8470 (0.7520-0.9420)
	Wet AMD	0.9695 (0.9061-1.0000)	0.9855 (0.9412-1.0000)	0.9828 (0.9348-1.0000)	0.9724 (0.9120-1.0000)	0.9772 (0.9220-1.0000)
	PM	0.9881 (0.9481-1.0000)	0.9484 (0.8673-1.0000)	0.9816 (0.9320-1.0000)	0.9751 (0.9176-1.0000)	0.9852 (0.9405-1.0000)

AUC: area under receiver operating characteristic curve; CI: confidence interval; DR: diabetic retinopathy; Dry AMD: dry age-related macular degeneration; ERM: epiretinal membrane; ME: macular edema; MIL: multiple instance learning; PM: pathologic myopia; Wet AMD: wet age-related macular degeneration.
